# Neuronal Assembly Detection and Cell Membership Specification by Principal Component Analysis

**DOI:** 10.1371/journal.pone.0020996

**Published:** 2011-06-15

**Authors:** Vítor Lopes-dos-Santos, Sergio Conde-Ocazionez, Miguel A. L. Nicolelis, Sidarta T. Ribeiro, Adriano B. L. Tort

**Affiliations:** 1 Brain Institute, Federal University of Rio Grande do Norte, Natal, Rio Grande do Norte, Brazil; 2 Edmond and Lily Safra International Institute of Neuroscience of Natal, Natal, Rio Grande do Norte, Brazil; 3 Duke University Center for Neuroengineering and Department of Neurobiology, Duke University, Durham, North Carolina, United States of America; University of Maribor, Slovenia

## Abstract

In 1949, Donald Hebb postulated that assemblies of synchronously activated neurons are the elementary units of information processing in the brain. Despite being one of the most influential theories in neuroscience, Hebb's cell assembly hypothesis only started to become testable in the past two decades due to technological advances. However, while the technology for the simultaneous recording of large neuronal populations undergoes fast development, there is still a paucity of analytical methods that can properly detect and track the activity of cell assemblies. Here we describe a principal component-based method that is able to (1) identify all cell assemblies present in the neuronal population investigated, (2) determine the number of neurons involved in ensemble activity, (3) specify the precise identity of the neurons pertaining to each cell assembly, and (4) unravel the time course of the individual activity of multiple assemblies. Application of the method to multielectrode recordings of awake and behaving rats revealed that assemblies detected in the cerebral cortex and hippocampus typically contain overlapping neurons. The results indicate that the PCA method presented here is able to properly detect, track and specify neuronal assemblies, irrespective of overlapping membership.

## Introduction

Hebb's seminal work constitutes a landmark of modern neuroscience [1]. His theory proposes detailed neural mechanisms for the processing and learning of information, from the molecular, cellular and circuit levels to the emergence of complex cognitive functions. According to Hebb's hypothesis, the recurrent co-activation of a subset of neurons would increase the efficiency of their connections, leading to the formation of a cell assembly. Therefore, synchronization of spike times would play a critical role in the creation of new assemblies [2], [3], [4], [5], [6]. In this context, a cell assembly is defined as a group of neurons that fire together and wire together. Due to the increased strength of the connections linking members of the assembly, activation of some of its neurons would trigger the activation of the entire neuronal group, leading to pattern completion [7], [8], [9]. Hebb also postulated that the activation of a cell assembly can lead to the sequential activation of other assemblies, a phenomenon he termed as phase-sequences, and proposed to underlie complex brain computations (see also [10], [11], [12]). In line with this view, neocortical and hippocampal information has been shown to be widely distributed over neuronal populations, rather than encoded by the activity of highly specialized cells [13], [14], [15], [16], [17], [18], [19].

The actual investigation of Hebbian cell assemblies and their dynamics is only beginning to be possible, thanks to major technological advances that allow the simultaneous and chronic recording of large neuronal populations [20], [21], [22]. In parallel with these advances, mathematical methods have been developed to address Hebb's hypotheses in experimental data, such as template matching of neuronal population activity [23], [24], [25] and the detection of precise multi-neuron firing [26], [27], [28], [29], [30]. Powerful methods for the detection of neuronal co-activation based on Principal Component Analysis (PCA) were also described [31], [32], [33], which have recently been extended to incorporate strong statistical support [34]. The latter framework is able to reliably detect the presence of cell assemblies and to assess ensemble activation with high temporal resolution based on the projection of network activity on the principal components (PCs) of the neuronal correlation matrix (see next section for a definition).

Despite its successful initial applications [32], [35], [36], the PCA-based method presents some limitations. First, it does not identify which specific neurons compose the detected assemblies. In addition, as demonstrated in the present work, the use of individual PCs in order to represent assembly activity patterns is misleading when there are neurons shared by different assemblies. As a consequence, in these cases the projection of neuronal activity based on PCs does not match the actual time course of individual assembly activation. Since it is currently believed that most, if not all, neocortical and hippocampal neurons take part in multiple assemblies (see Discussion), such limitation is an important one.

To address these gaps, we present here an exploration of some of the key properties of the PCA method for assembly detection, and propose critical modifications of the current framework. First, we show that the number of assemblies and assembly neurons can be computed from the analysis of the eigenvalues of the neuronal correlation matrix. We then show that the subspace spanned by the PCs can reveal which neurons compose the detected assemblies. We go on to show how the time course of the activity of individual assemblies can then be estimated, even when different cell assemblies have a subset of common neurons. Finally, we show that our method can properly detect, track and specify the neuronal membership of neocortical and hippocampal assemblies recorded from behaving rats.

## Results

**Figure 1:**
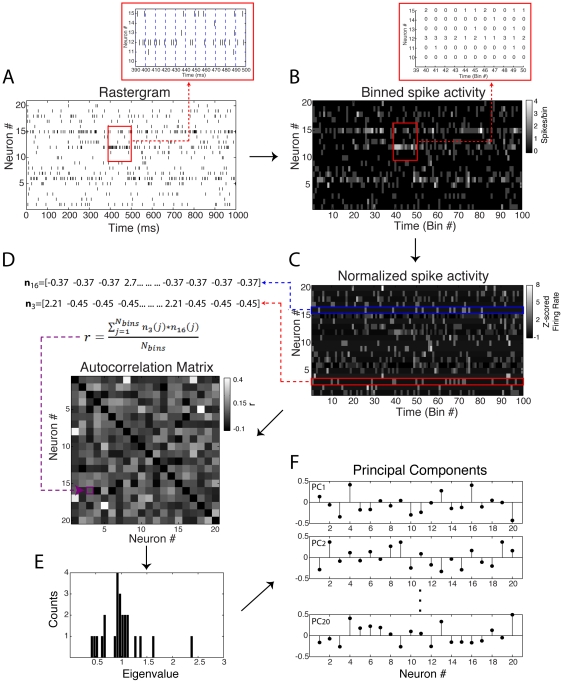
Original method overview. (**A**) Raster plot activity. Each row represents a neuron; marks denote an action potential and *x*-axis represents time. Panel inset shows the binning procedure into non-overlapping time windows. (**B**) Binned spike activity matrix obtained from raster plot in **A**. Each element is the count of the number of spikes in a given bin. (**C**) Z-scored binned spike activity matrix obtained by mean and variance normalization of the matrix in **B**. (**D**) Autocorrelation matrix (ACM) of the normalized binned spike activity in **C**. Each element denotes the linear correlation between two neurons. The main diagonal is set to zero for clearer visualization. (**E**) Eigenvalue histogram of the ACM shown in **D**. (**F**) Principal components (PCs) of the ACM, which are the eigenvectors associated with the eigenvalues shown in **E**. PCs are ordered in respect to their eigenvalues, i.e., the PC1 is associated with the highest eigenvalue and so on.

First we briefly outline the general framework as proposed in [31], [32], [34], [35]. Figure 1A shows an example of neuronal population activity represented by means of a standard spike rastergram plot, in which each mark denotes the firing of an action potential by a neuron (the y-axis indicates the neuron labels). The procedure begins by binning the spike rastergram into non-overlapping, short time windows (referred to as bins) and counting the number of spikes in each bin, as indicated in Figure 1B. In this way, the rows of the resulting matrix represent neuronal units, and the columns represent the time bins. More specifically, the element *a_ij_* denotes the number of spikes of the i*th* neuron in the j*th* bin (Figure 1B inset). For the sake of generality, in this work we use the “bin number” (“bin #”) as our arbitrary unit of time. Next, the binned spike activity is z-scored in order to normalize the spike rate of each neuron (Figure 1C). Thus, the rows of the normalized matrix are vectors with zero mean and unit variance. The autocorrelation matrix of the normalized spike activity is then computed (Figure 1D); each entry *ij* of the autocorrelation matrix is the Pearson correlation coefficient (*r*) between the rows *i* and *j* of the matrix shown in Figure 1C (i.e., a correlation between two spike rate vectors).

**Figure 2:**
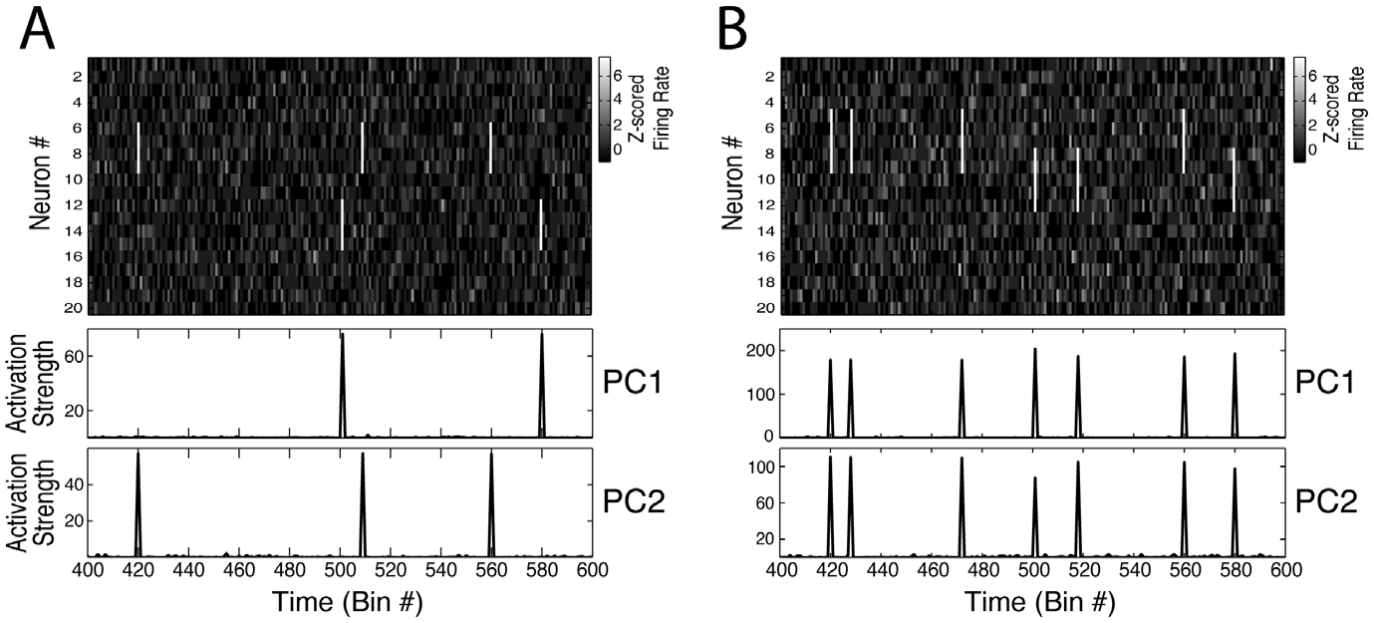
PCs do not always isolate the activity of different cell assemblies. (**A**) Top panel shows a binned spike activity matrix with 20 neurons (modeled as Poissonian processes) and 8000 time bins. Two cell assemblies were simulated in the network, each having four neurons (Assembly 1 neurons: #6, #7, #8, #9; Assembly 2 neurons: #12, #13, #14, #15). Neurons in the same assembly were set to fire together six times above their mean firing rate at 0.5% of the bins. Bottom panels show the estimated time course of ensemble activity obtained by the projection of the binned spike activity using the projector operator defined as the outer product of the PCs (see Methods). Note that PC1 marks the activations of Assembly 2, and PC2 marks the activations of Assembly 1. (**B**) Same as **A**, but with assemblies sharing neurons (Assembly 1 neurons: #5, #6, #7, #8, #9; Assembly 2 neurons: #8, #9, #10, #11, #12). Note that for this example this framework fails to isolate the activity of individual assemblies.

The next steps of the method involve the computation of the eigenvalues of the autocorrelation matrix (Figure 1E) and the associated eigenvectors (Figure 1F), which in this context are referred to as Principal Components (PCs). Finally, the PCs associated with significant eigenvalues (see below) are used to track the activity of cell assemblies in each time bin (Figure 2A).

An important question is to know when the correlation coefficient of the spike activity of two neurons can be considered statistically significant for a given dataset. To this end, a statistical threshold that separates non-significant correlations from values above chance is needed. Instead of using exhaustive surrogate methods [37], [38], [39], [40], [41], [42], Peyrache et al. elegantly addressed this problem by analyzing the distribution of the eigenvalues of the autocorrelation matrix [34], [35]. From random matrix theory, it can be demonstrated that the eigenvalues of an autocorrelation matrix computed from a matrix with statistically independent rows (in our case, neurons with independent activity) follow the so-called Marčenko-Pastur distribution [43]. Since the goal is to identify ensemble activity, i.e. groups of neurons with correlated firing, the theoretical upper limit provided by the Marčenko-Pastur distribution can be used as statistical threshold. Thus, if there are groups of significantly correlated neurons in the population recorded, some eigenvalues will lie above this statistical threshold. Furthermore, the PCs associated with significant eigenvalues can be used to track assembly activity. This is accomplished by projecting the normalized spike activity matrix using projector operators computed from the PCs, resulting in a unidimensional signal representing the time series of ensemble activity.

Using simulated data, we show in Figure 2A that the activation time course computed as described above is able to represent the activity of specific cell assemblies in some cases. However, as shown in Figure 2B, this approach is unable to separate the activity of individual assemblies when the neuronal population is composed of assemblies with overlapping cells. Note in Figure 2A that the estimated time courses of the activation strength correspond to increases of firing rate of specific subsets of neurons, as desired. However, for the case depicted in Figure 2B, the projection of population activity using the PCs does not separate the activity of the two cell assemblies. This constitutes an important limitation since the existence of assemblies with shared neurons is expected (see Discussion).

In the following sections we explore in more detail the general characteristics of this method, and propose modifications to allow tracking the activity of individual assemblies even when they share neurons. We also show that it is possible to precisely identify the neurons participating in each cell assembly.

### Marčenko-Pastur distribution and the null hypothesis of independent neuronal activity

**Figure 3:**
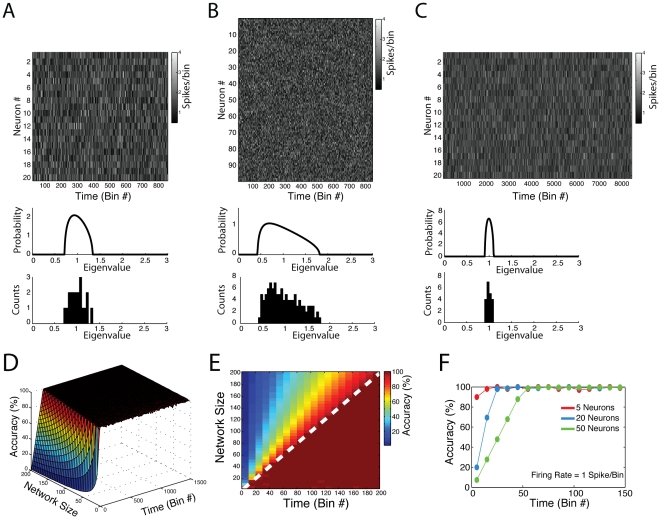
Eigenvalues of autocorrelation matrices derived from the activity of independent neurons fall within theoretical bounds. (**A**) Top Panel: Binned spiking activity of 20 independent neurons. Each neuron was simulated as following a Poisson process (mean = 1 spike/bin). Middle Panel: Theoretical Marčenko-Pastur distribution. Bottom Panel: Histogram of eigenvalues obtained from the autocorrelation matrix computed from the neuronal activity shown in the top panel. (**B,C**) Similar panels as in **A** but for network activities presenting a greater number of neurons (**B**) or bins (**C**). Notice that the eigenvalues follow the Marčenko-Pastur distribution in all cases, and that the width of the predicted distribution is dependent on the ratio *N_n_*_eurons_/*N*_bins_, where *N_n_*_eurons_ = number of neurons and *N*_bins_ = number of bins. (**D**) Percentage of eigenvalues falling within Marčenko-Pastur theoretical bounds as a function of network size and number of time bins. For each parameter set, neurons were simulated as independent Poisson processes (mean = 1 spike/bin). Values represent the mean over 20 simulations. (**E**) Top-down view of the surface in **D**. Notice that virtually 100% accuracy occurs when *N*_bins_>*N_n_*_eurons_. Dashed white line denotes *N*_bins_ = *N_n_*_eurons_. (**F**) Transections of the surface in **D** obtained for three different network sizes.

We start by exploring example cases of networks where no organized neuronal activity is present, that is, when there is no cell assembly in the network. As already introduced in the previous section, the eigenvalues of an autocorrelation matrix computed from a matrix with independent rows follow the Marčenko-Pastur distribution (see Methods for its formula). In order to illustrate this prediction, we show in Figure 3A–C three examples of random network activity differing in the number of neurons and time windows analyzed (i.e., the total number of bins). Each neuron is modeled as an independent Poissonian process (mean = 1 spike/bin). The predicted distribution of eigenvalues is shown below the corresponding network along with its empirical eigenvalues histogram. As expected, the actual eigenvalues follow the Marčenko-Pastur distribution. Note that the theoretical distribution has lower variance for greater values of the ratio *q* = *N*_bins_ */N*_neurons_.

We next performed a systematic parametric study of matrices with independent rows to investigate this property further. To this end, we defined “accuracy” as the percentage of eigenvalues that lie within theoretical bounds, that is, 100% accuracy means that all eigenvalues are within the limits predicted by the Marčenko-Pastur distribution. In other words, accuracy assesses the performance of the use of the theoretical bounds in determining the absence of cell assemblies in the network.

Figure 3D shows accuracy as a function of network size and total number of bins. Notice that, for a given network size, higher levels of accuracy are achieved with a higher number of time bins. In fact, as better seen in Figure 3E, accuracy is highly dependent on the condition *q* = *N*_bins_ */N*_neurons_>1, i.e., the number of analyzed bins has to be greater than the number of neurons in the network. Figure 3F displays the results shown in Figure 3D for three specific network sizes. Similar results were obtained for different firing rates and also for the more realistic case in which the mean firing rate of each neuron differs from the mean rate of other neurons (data not shown). This latter result was expected since the firing rates are normalized.

Overall, we conclude that the theoretical limits predicted by the Marcenko-Pastur distribution can be used as the null hypothesis of independent neuronal activity, as long as the number of bins analyzed is higher than the number of neurons in the network. In the next section, we show how we can also use this theoretical distribution to determine the precise number of cell assemblies in the network.

### Eigenvalues outside theoretical bounds mark the number of cell assemblies and assembly neurons

We have shown above that eigenvalues of autocorrelation matrices computed from independent neuronal activity remain within predicted limits as long as the condition *q*>1 is satisfied. Now we go further to show that the number of eigenvalues above the theoretical upper limit not only indicates the presence of ensemble activity, but it is also an accurate estimation of the number of cell assemblies in the network.

**Figure 4:**
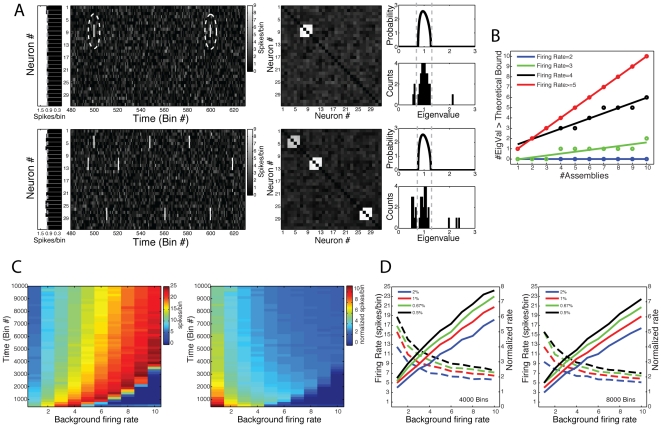
Eigenvalues above theoretical bound mark the number of cell assemblies. (**A**) *Top*: (Left panels) Shown are binned spiking activity of a network composed of 32 neurons (second panel), along with the average firing rate of each neuron (first panel). Total simulation time was 8000 bins; neurons were modeled as possessing a Poissonian firing rate (mean = 1 spike/bin). In order to simulate a cell assembly, we set a group of neurons to activate simultaneously at 0.5% of the bins (firing rate within activation events = 6–9 spikes/bin). To facilitate visual inspection, neighbor neurons were chosen as composing the cell assembly (neurons #7, #8, #9, #10; dashed circle). (Middle Panel) Network correlation matrix. Notice a cluster of correlated activity corresponding to the cell assembly. (Right Panels) Theoretical eigenvalues distribution for independent neuronal activity (top panel), and the eigenvalues histogram computed from the simulated network (bottom panel). Notice that 1 eigenvalue lies above the theoretical upper limit predicted for random activity. *Bottom*: Same as above, but for a network presenting three cells assemblies (Cell assembly 1: neurons #3, #4, #5, #6; Cell assembly 2: #10, #11, #12, #13; Cell assembly 3: neurons #26, #27, #28, #29). Notice that three eigenvalues lie above the theoretical bound. (**B**) Number of eigenvalues above the theoretical bound as a function of the number of cell assemblies in the network for different values of firing rate during cell assembly activation events. Networks were composed of 40 neurons; neurons were simulated as Poissonian processes (background mean = 1 spike/bin). Total simulation time was 8000 bins; assembly activation frequency was set to 0.5% of the bins. Each cell assembly was composed by 4 neurons (non-overlapping). Colored lines denote the linear fit *y* = α+β*x* for each activation firing rate studied. Notice that the higher the firing rate within activation bins, the higher the slope coefficient (β). If the firing rate is high enough, β equals 1, which characterizes the regimes in which the number of eigenvalues perfectly corresponds to the number of cell assemblies. Each data point represents a single simulation result. (**C**) Pseudocolors denote the minimal firing rate within activation bins leading to β equal to 1 as a function of the background mean firing rate (x-axis) and total number of time bins (y-axis). Results are expressed as absolute values (left) and as a ratio relative to the background firing rate (right). Assembly activation frequency was set to 0.5% of the bins. Networks were composed by 40 neurons, and each cell assembly was composed by 10 neurons. For each parameter set, values represent the mean over 20 simulations. (**D**) Left panel: Black line represents a transection of the result in **C** for network activities of 4000 time-bins. Other colored lines represent equivalent results obtained for different frequencies of cell assembly activation, as labeled. Notice that the higher the frequency of cell assembly activation, the lower the minimal firing rate leading to β equal to 1. Colored dashed lines represent the same result but as a ratio to the background firing rate. Right panel: Similar results as before, but for a network activity composed of 8000 time-bins.

In Figure 4A two examples of neuronal network activity are shown. Neurons were modeled as Poissonian processes as in Figure 3, but, in addition, simulated assembly activity was added to the network. Assembly activations were modeled as an increase of the firing rates of a subset of neurons in specific bins. More specifically, in these “activation bins”, neurons were set to fire between 6 and 9 spikes, uniformly distributed. Both examples have 32 neurons and 8000 time bins. In each example, the mean firing rates (over all time bins) of cell assembly neurons were not necessarily higher than those of the other neurons in the network (Figure 4A, leftmost panels). In other words, the specific bins of assembly activation did not lead to a considerable net change in the average spike frequency of these neurons. In Figure 4A we depict a period of 150 bins in which assembly activations can be seen (second panels from left), along with the autocorrelation matrix of the simulated network (third panels from left); the theoretical eigenvalue distribution and the empirical eigenvalue histogram are also shown (top and bottom rightmost panels, respectively).

In the first example, a cell assembly with four neurons (neurons #7, #8, #9 and #10) is present in the network. Neurons have independent activity, with the exception of the cell assembly neurons that have higher firing rate in 0.5% of the bins randomly selected (i.e., the activation bins; cell assembly neurons have independent activity in the other bins). A simple visual inspection of the autocorrelation matrix already reveals higher correlations among cell assembly neurons. Importantly, notice that one eigenvalue of the empirical distribution lies above the upper limit predicted for independent neuronal activity in this example. In the second example, three cell assemblies were added to the network. Notice that three eigenvalues fall above the theoretical upper limit in this case. These results therefore suggest that the number of eigenvalues above the Marcenko-Pastur distribution mark the number of cell assemblies in the network. We next performed a parametric analysis to investigate in more detail such property.

In Figure 4B, we analyze networks with different numbers of assemblies and different firing rates during activation bins (“activation firing rate”). We simulated networks with 40 neurons (mean spike rate = 1 spike/bin) and 8000 time bins; assemblies were composed by 4 neurons and set to be active in 0.5% of the bins. Each data point in Figure 4B corresponds to a network with a given level of activation firing rate (labeled by colors) and number of assemblies (varying from 1 to 10, as indicated in the x-axis). The number of eigenvalues above the theoretical upper limit is plotted as a function of the number of assemblies for different activation firing rates. Note that a perfect match between the number of eigenvalues above the upper limit and the number of assemblies in the network is indicated by β = 1 in the linear fit *y* = α+β*x*. We found that the number of eigenvalues above the upper limit underestimated the number of assemblies in the network (β<1) in cases in which assembly activations had a firing rate below 5; on the other hand, all cases with activation firing rate equal or above 5 presented a perfect match (β = 1). Taken together, these results suggest that a minimal activation firing rate is required for the proper detection of the number of assemblies.

Our next step was to conduct exhaustive simulations to investigate the requirements for reaching the criterion β = 1. Figure 4C shows the minimal assembly activation firing rate required to achieve such criterion as a function of background firing rate and number of analyzed bins. In the left panel, the minimal activation firing rate is shown in absolute values, while in the right panel it is expressed as a ratio relative to the background firing rate. Note that for a higher number of bins analyzed, a lower activation firing rate is required for a perfect match between the number of assemblies and of the eigenvalues above the upper limit. Figure 4D illustrates the dependence of β = 1 on the number of assembly activation events. We studied network activities with 4000 (Figure 4D left panel) and 8000 (Figure 4D right panel) bins for four different “activation frequencies” (number of activation bins/number of time bins), and we show the minimal activation firing rate for β = 1 as a function of background activity. Notice that, as the activation frequency gets higher, lower assembly activation firing rates are sufficient for β = 1. Overall, these simulations show that the number of eigenvalues above the theoretical bound is related to the number of assemblies present in the network. The efficiency of such estimation depends on how many bins the assembly neurons are correlated and how high this correlation is.

**Figure 5:**
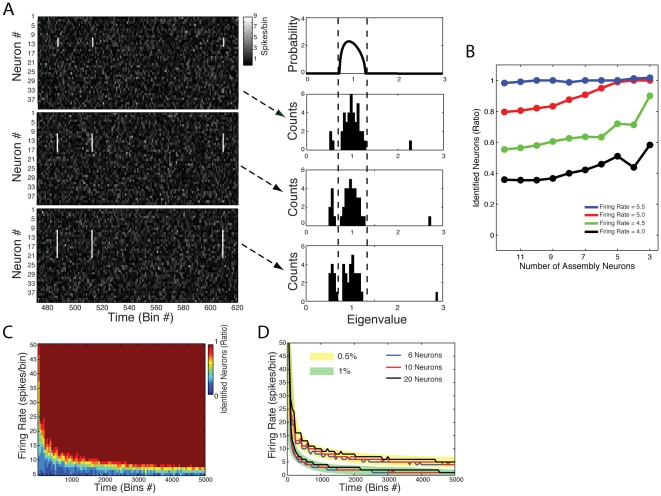
The number of eigenvalues lying outside the theoretical distribution limits corresponds to the number of cell assembly neurons. (**A**) Shown are the binned spiking activity matrices of networks composed of 40 neurons (left panels), along with the predicted eigenvalues distribution for independent neuronal activity (top right panel) and the actual eigenvalue histogram (bottom right panels). Total simulation time was 8000 bins; neurons were modeled as possessing a Poissonian firing rate (mean = 1 spike/bin). The 3 cases depicted differ in the number of neurons that compose the cell assembly. Notice that, for all cases, the number of eigenvalues outside the theoretical limits (dashed lines) matches the number of neurons in the cell assembly. (**B**) Ratio of the number of eigenvalues outside theoretical limits to the number of cell assembly neurons (ratio = 1 means that the number of significant eigenvalues perfectly corresponds to the number of cell assembly neurons). Different data points denote the mean over 20 simulations for different number of cell assembly neurons (x-axis) and activation firing rates (colored lines), as labeled. Networks were composed of 40 neurons; neurons were simulated as Poissonian processes (background mean = 1 spike/bin). Total simulation time was 8000 bins; assembly activation frequency was set to 1% of the bins. (**C**) Pseudocolors denote the ratio of the number of eigenvalues outside theoretical limits to the number of cell assembly neurons as a function of the activation firing rate and total time bins. Values represent the mean over 20 simulations. Networks were composed of 40 neurons; the cell assembly was made of 10 neurons set to activate at a frequency of 0.5% of the bins. Notice that for each activation firing rate, a perfect estimation of the number of cell assembly neurons (ratio = 1) is achieved if the number of bins analyzed is large enough. (**D**) Minimal activation firing rate required for a perfect match between the number of eigenvalues outside predicted limits and the number of cell assembly neurons as a function of the number of analyzed bins. Different lines represent different cases varying in the number of neurons in the assembly and in the frequency of cell assembly activation, as labeled.

**Figure 6:**
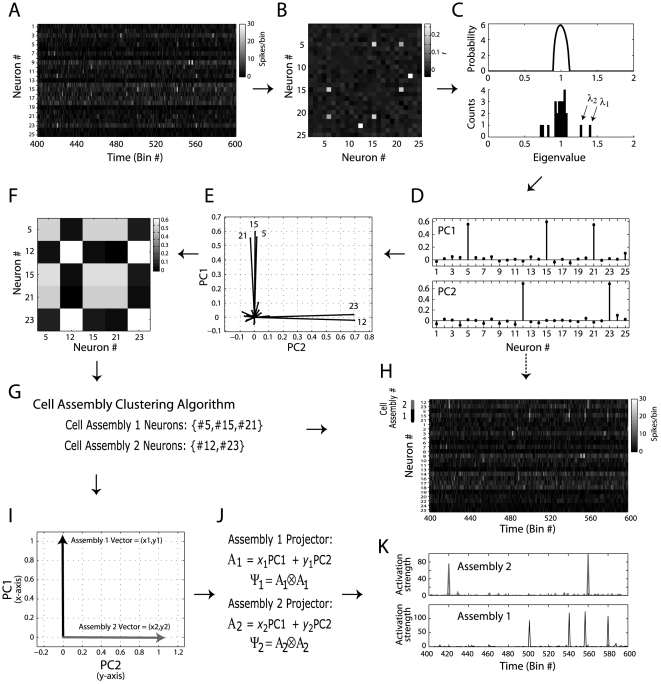
Principal component-based analysis identifies cell assembly neurons and the time course of their activation. (**A**) Binned spiking activity of a network composed of 25 neurons simulated for 8000 bins (200 bins shown). Neurons are modeled as Poissonian processes with random mean rate between 1 and 5 spikes/bin, uniformly distributed across the neurons. In addition, each neuron is set to fire at 6× its mean rate at 0.5% of the bins randomly chosen (referred to as activation bins). In order to simulate cell assemblies, we set all activation bins to be independent, except for two groups of neurons which have simultaneous activation bins. (**B**) Autocorrelation matrix (ACM). (**C**) Theoretical eigenvalues distribution for independent neuronal activity (top), and the eigenvalues histogram computed from the simulated network (bottom). Note that 2 eigenvalues fall above the theoretical upper limit predicted for random activity, which correspond to the two cell assemblies present in the network. Notice further that three other eigenvalues fall below the lower limit; the number of eigenvalues outside the theoretical limits is therefore 5, which corresponds to the number of neurons participating in cell assemblies. (**D**) ACM eigenvectors associated with the two eigenvalues above the theoretical limit for random activity. These vectors are referred to as principal components (PCs). (**E**) Neuronal representations in the subspace spanned by the PCs (referred to as the Assembly Space). Since the PCs are the vectors which best describe strong correlated activity, neurons with larger projections on the Assembly Space are the neurons involved in cell assemblies (the label of these neurons are also shown). (**F**) Interaction Matrix; the entries of this matrix are measures of correlated activity of cell assembly neurons in the Assembly Space. Higher values denote neuron pairs pertaining to the same cell assembly, whereas lower values denote neurons whose activity is orthogonal. (**G**) From the Interaction Matrix, a simple clustering algorithm (described in Supplementary Information files) identifies the neurons of each cell assembly. (**H**) Same binned spiking activity as in **A** but rearranged in order to show cell assembly neurons on top, as labeled. (**I,J**) Assembly Vectors are defined as mean vectors in the Assembly Space (**I**); these vectors are used to compute projector operators (**J**). (**K**) The projector operators are then applied to the binned spiking activity, revealing the time course of the activation strength of each cell assembly. Note that these results corroborate the activations seen by visual inspection of **H**. Since the cell assemblies were non-overlapping in this example, the identity of cell assembly neurons can be directly inferred by a simple analysis of the PCs (represented by the dashed line from **D** to **H**). However, such straight inference cannot be performed in cases where one or more neurons pertain to two or more assemblies (see Figure 7).

**Figure 7:**
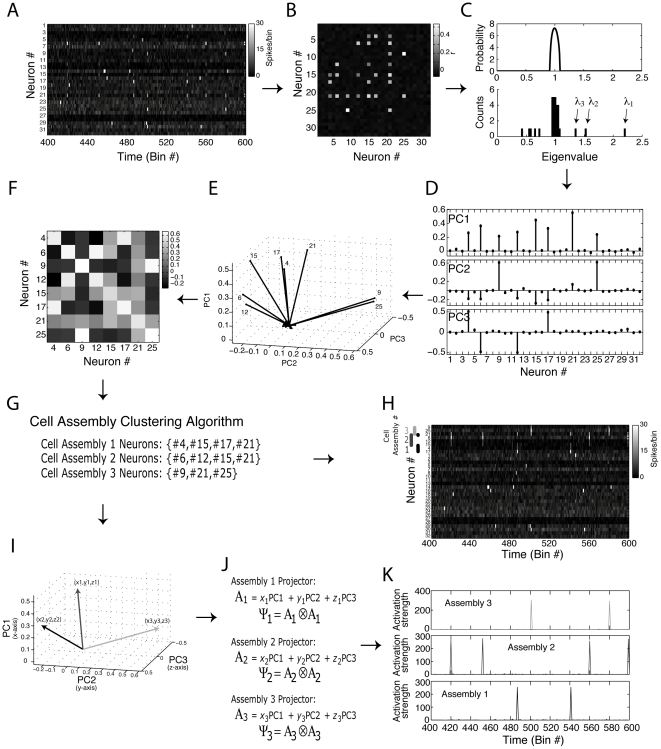
Identification of cell assemblies with overlapping neurons. (**A–K**) Same panels as in Figure 6, but for a network composed of three cell assemblies presenting common neurons. See text for further details.

Next, we studied the eigenvalues that fall below the lower theoretical bound. Inspection of Figure 4A suggests that the number of eigenvalues below the predicted limit for independent activity increases when more assemblies are added to the network. In Figure 5, we show that, in fact, the total number of eigenvalues outside the theoretical distribution (below or above) is a good estimation of the total number of neurons involved in ensemble activity. More specifically, in Figure 5A we show three examples of networks with 40 simulated neurons and 8000 analyzed bins. A cell assembly was added to the network (active in 0.5% of the bins) and the number of neurons composing the ensemble was varied (4, 8 and 12 assembly neurons from top to bottom panels). The eigenvalue histograms shown in Figure 5A indicate that the number of eigenvalues below the predicted limit increases with increasing the number of cell assembly neurons; in fact, for the 3 examples, the total number of eigenvalues outside the theoretical distribution perfectly matched the number of cell assembly neurons. In Figure 5B we show that this property depends on the assembly activation firing rate. We again used exhaustive simulations in order to assess the robustness of this estimation. Figure 5C shows the number of eigenvalues outside predicted limits as a function of assembly activation firing rate and analyzed bins; the result is expressed as a ratio of the number of the neurons composing the assembly (# outer eigenvalues/# assembly neurons). Note that a virtually perfect estimation (ratio = 1) is approached as the activation firing rate and the number of analyzed bins increase. Figure 5D shows the minimal activation firing rate for ratio = 1 as a function of the number of analyzed bins. We show this relation for different assembly activation frequencies and for different assembly sizes. While the estimation does not depend significantly on the number of neurons in the assembly, it is improved if the assembly is active in more bins. Similar findings were obtained in networks composed by multiple assemblies, even when some neurons were shared by two or more assemblies (simulations not shown, but see Figures 6 and 7).

In conclusion, we observed that the empirical distribution of eigenvalues not only indicates the presence of ensemble activity in the network, but can also be used to estimate the number of cell assemblies present in the network as well as the number of neurons involved in ensemble activity. In the next section we show how this information can be used to identify which neurons belong to each detected assembly.

### Identification of cell assemblies and time course of their activation

So far we have shown that eigenvalues of autocorrelation matrices that are higher than a well-established statistical threshold have a strong relation with subsets of correlated neurons. Since these eigenvalues are by definition associated with PCs, it is reasonable to expect that these vectors also carry information about ensemble activity. In order to show how they can be used to identify assemblies in a network (in terms of which neurons compose them) we created a simulated network as an illustrative example. Neurons were again modeled as Poissonian processes, but with different mean spike rates (uniformly distributed between one and five spikes/bin). In addition, we set every neuron to fire 6 times above their mean rate at 0.5% of the bins. Two groups of neurons (cell assembly 1 neurons: #5, #15, #21; cell assembly 2 neurons: #12, #23) had these firing peaks at the same bins, simulating assembly activations; non-assembly neurons had peak firing at independent (randomly chosen) bins.

Figure 6A shows a 200-bin interval of the simulated network; the associated autocorrelation matrix is shown in Figure 6B. Two eigenvalues of this matrix fall above the upper theoretical limit, whereas three eigenvalues lie below the lower bound (Figure 6C). This analysis therefore indicates that two assemblies and a total of five assembly neurons are present in the network, consistent with predefined simulation parameters.

Since eigenvalues above statistical threshold represent ensemble activity, we use the PCs associated with them (Figure 6D) to search for the identity of assembly neurons. The autocorrelation matrix can be seen as 25 vectors in a 25-dimensional space. In this case, PCA roughly means that the detected assembly activity is better described by the subspace spanned by the PCs; in the present work, we refer to this subspace as “Assembly space”. Removing the non-principal components of our analysis is equivalent to filtering the autocorrelation matrix in order to unravel assembly activity.

Figure 6E shows the neuron vectors on the Assembly space, which are obtained straight from the PC entries (see Methods). Note that some neurons present large vector length in this space, indicating that their spike activity is related to the detected assemblies. In fact, the five neurons with large vector length in the Assembly space (labeled in Figure 6E) correspond to the five units participating in assembly activity. Notice further that there are two clusters of neuron vectors in the Assembly space; these clusters are roughly orthogonal to each other, indicating independent activity. Indeed, notice that neurons orthogonal to each other pertain to different assemblies. Thus, we computed the length of the projection of each neuron vector onto the direction of the others and expressed these results in an “Interaction Matrix” (Figure 6F; see Methods). From the Interaction matrix, we used a simple clustering algorithm in order to determine which neurons were in each assembly (Figure 6G). Although the identification of assembly neurons was straightforward in this example from the visual inspection of Figure 6F, we noted that this was not always the case, making the use of a robust algorithm necessary (see Methods and Figures S1 and S2 for details about the algorithm). Figure 6H shows the same binned spike activity as in Figure 6A but with rows reordered with respect to the identified assemblies. Note that neurons within an assembly have firing peaks at the same bins.

The use of PCs was previously proposed in order to create projectors for computing ensemble activity with a single bin resolution [31], [32], [34], [35]. An activity projector can be defined as the outer product of a PC with itself ([34]; see Methods for details). Since each PC represents an activity pattern, it is possible to compute the instantaneous strength of this pattern by multiplying the z-scored binned spike activity with the projector derived from the PC (see Methods). However, as shown in Figure 2, in some cases this method does not represent individual assemblies. To overcome this limitation, we propose another vector to construct the projectors. This vector, called “assembly vector”, is defined as the mean over all neuron vectors in the Assembly space that exclusively pertain to a given assembly (Figure 6I). Notice that the assembly vector is a linear combination of the PCs (Figure 6I,J), which allows obtaining this vector in the 25-dimensional space. By using this optimal assembly vector to construct the activity projector, we were then able to obtain the time course of the activity of the corresponding cell assembly. Figure 6K shows the results of such approach. For each assembly, the peaks of the time course matched perfectly the assembly activations seen in Figure 6H.

Note that in this example the PC weights directly reveal the neurons composing each assembly (Figure 6D). For instance, PC1 had higher values in dimensions 5, 15 and 21, which correspond to Cell Assembly 1 neurons; by the same token, the high values of PC2 denote Cell Assembly 2 neurons. Consequently, the estimated assembly optimal vectors in Figure 6I are very similar to the PCs and thus the activity projectors computed from the assembly vectors are virtually the same as the ones calculated from the PCs. As already mentioned (see Figure 2), the previous framework is able to track individual assembly activity when there are no overlapping neurons among the assemblies, as is the case of the example shown in Figure 6; therefore, our modified approach is equivalent to the original in these cases (see Figure S3).

In Figure 7 a more complex example is shown. The network activity was modeled as in Figure 6, but with three assemblies present in the network. Moreover, we simulated overlapping neurons between the assemblies (Assembly 1 neurons: #4, #15, #17, #21; Assembly 2 neurons: #6, #12, #15, #21; Assembly 3 neurons: #9, #21, #25). Figure 7C shows that 3 eigenvalues lie above the upper theoretical limit, denoting the three cell assemblies; moreover, the number of eigenvalues outside the theoretical limits matches the number of cell assembly neurons (8 in this example). Note in Figure 7D that it is no longer possible to identify the assemblies (in terms of which neurons compose them) by a visual inspection of the PC weights. Therefore, the estimation of the time course of assembly activity by computing the projectors from the PCs would be misleading in this case (see Figure S3).

As in the former example, projecting the neuron vectors on the Assembly space reveals the cell assembly neurons (Figure 7E). Note that the assembly neurons are not clearly clustered as in the example shown in Figure 6. While neurons that only pertain to the same assembly still tend to cluster together, neurons that participate in more than one assembly cannot be in two clusters simultaneously. For instance, projected neuron #15 is orthogonal to projected neurons #9 and #25. This is because neuron #15 does not compose the assembly in which neurons #9 and #25 participate. Conversely, neuron vector #15 is not orthogonal to any of the other neuron vectors, since they all participate in at least one assembly together with neuron #15. That is, overlapping neurons still have relatively large degree of collinearity with neurons that compose the same assemblies (Figure 7F). In this sense, since neuron #21 is in all assemblies, it is not orthogonal to any other assembly neuron.

As in the former example, pairwise relations between neurons in Assembly space can be inferred from the Interaction matrix. Notice however that in this case it is not straightforward to identify the cell assemblies by visual inspection of the Interaction Matrix (Figure 7F). Nevertheless, the clustering algorithm we developed (see Methods and Figures S1 and S2) was able to identify the precise composition of each assembly (Figure 7G). As before, after identifying the assemblies we computed the optimal assembly vectors (Figure 7I,J) and used them to project the proper time course of assembly activations (Figure 7K; compare with Figure S3). This example therefore shows that the use of assembly vectors instead of PCs is better suited for computing assembly activity.

### Examples of applications to real data

So far we have used simulations to introduce a PCA-based method for cell assembly detection, providing details about how each step worked. In this section we apply the framework to real data and further compare the modifications we propose with the original method.

**Figure 8:**
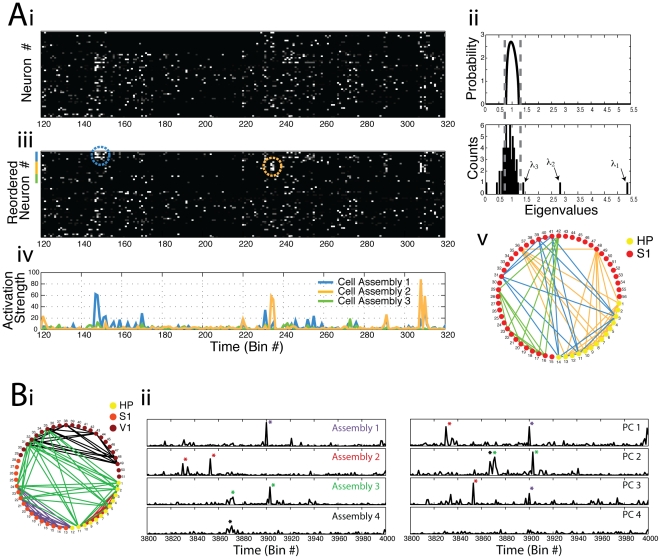
Example of cell assembly identification using principal components in an experimental data-set. (**A**) Ai: Binned spiking activity for 14 hippocampal and 42 S1 neurons obtained from a rat during exploration of a novel object (see Ribeiro et al. [44]). Bin size = 30 ms; total time analyzed: 117.51 s. Aii: Theoretical eigenvalues distribution for independent neuronal activity (top) and the eigenvalues histogram computed from the actual network (bottom) exhibiting 3 eigenvalues above the theoretical upper limit predicted for random activity. Aiii: Same binned spiking activity as above, but with reordered rows such that neurons pertaining to cell assemblies are displayed in the top rows (color bars near the top of the y-axis mark cell assembly neurons; colored dashed circles highlight example periods of assembly activation). Aiv: Projection analysis yielding the activation time course for the three cell assemblies indentified in this network (notice that cell assembly 3 does not activate in the period shown). Av: Graph diagram showing detected cell assemblies (connected neurons). Notice that inter-regional cell assemblies are revealed. (**B**) Bi: Graph diagram showing four assemblies detected in recordings from S1, V1 and hippocampus (HP) during slow-wave sleep (Bin size = 30 ms; total time analyzed = 124 s). Bii: Time course of ensemble activity as estimated by the original (right) and modified (left) framework.

We analyzed spike activity recorded from rats chronically implanted with multielectrode arrays (see Methods). In the first example (Figure 8A), neuronal activity was obtained from the hippocampus and primary somatosensory cortex (S1) during the exploration of novel objects [44]. In Figure 8Ai, we show a 200-bin period (bin size = 30 ms) of spike activity of this network; Figure 8Aii shows the Marčenko-Pastur distribution along with the empirical eigenvalue distribution computed from the associated autocorrelation matrix. Note the detection of three assemblies in this example. We then applied the framework described above, and in Figure 8Aiii we plot the reordered spike activity with respect to the assemblies; dashed circles depict two examples of assembly activations occurring in the time period displayed. Figure 8Aiv shows the time course of activation of the detected assemblies; notice that the activation peaks match the activations seen in Figure 8Aiii. Finally, in Figure 8Av we show all neurons in a circular grid (hippocampal neurons: #1–14; S1 neurons: #15–56) and represent the assemblies by colored lines. Notice that our modified method allows us to infer that two assemblies have neurons in both brain regions.

In the second example (Figure 8B), we analyzed neurons recorded from the hippocampus, S1 and primary visual cortex (V1) during slow-wave sleep (hippocampal neurons: #1–12; S1 neurons: #13–28; V1 neurons: #29–51). Analysis of the eigenvalues revealed that 4 cell assemblies were present in this network (not shown). We again applied our framework to get to the precise identity of the assembly neurons and depict the four assemblies in Figure 8Bi. Notice in this panel that one assembly was composed by neurons from the three brain areas, whereas three other assemblies were restricted to a single brain region. Notice further in this example that some neurons participate in two assemblies. We then compared the time course of ensemble activity when the PCs were used to build the projectors with projectors derived from assembly optimal vectors. The left and right panels in Figure 8Bii show the activity time course estimated by the assembly vector and by the direct use of the PCs, respectively. Note that the individual assembly activations estimated by the assembly vector approach appear mixed in different PC projections. For instance, the PC1 projection carries mixed activations of Assemblies 1 and 2, whereas PC2 carries information about Assemblies 3 and 4. Based on these results and the simulations presented above, we conclude that the use of assembly vectors to compute the activity time course is well suited for discriminating the activation of individual assemblies, even in the presence of overlapping neurons.

## Discussion

We have presented a mathematical method for the identification of cell assemblies and for computing their activity as a function of time (in units of time bins). The overall algorithm is based on PCA and can be divided in three major steps: (**1**) *Detection of the number of cell assemblies and assembly neurons*; (**2**) *Identification of cell assemblies*; and (**3**) *Computation of assembly activity as a function of time*. The algorithm presented here constitutes an extension of powerful methods introduced previously [31], [32], [34]. The adaptations and extensions we propose make our framework able to circumvent important limitations present in former methods.

With respect to step 1, Peyrache et al. introduced the use of the Marčenko-Pastur distribution as the null hypothesis to determine the presence of ensemble activity [34]. This is an important achievement in terms of computational cost because most of the previous methods relied on surrogate data analyses to determine statistical significance [26], [30], [45], [46], [47], [48], [49]. Moreover, other methods are only feasible for a small number of neurons [29], [30], [50], [51] or only analyze pairwise correlations [45], [52], [53], making the analysis of large networks troublesome. The framework presented here inherits the computational advantages of the method envisioned by Peyrache et al. [34]. Additionally, it provides a clear interpretation for the eigenvalues derived from autocorrelation matrices of neuronal spike activity and their relation to the Marčenko-Pastur distribution: we showed that the number of eigenvalues significantly different from the random distribution contain useful information about the number of assemblies and the number of neurons participating in cell assemblies. This constitutes step 2 in our framework, which was not present in previous formulations.

Regarding step 3, the use of PCs in order to construct a time series of ensemble activity had already been introduced by Nicolelis et al. [31], [32]. More recently, Peyrache et al. [34] proposed the use of projectors computed from the PC vectors associated with significant eigenvalues to extract *patterns of neural activity* from a defined *template* epoch to be later assessed in a *match* epoch. Peyrache and colleagues used this approach to obtain ensemble activation signatures from spike activity of medial prefrontal cortex (mPfc) neurons during a learning stage (*template* epoch). Next they used these operators to measure instantaneous similarities (i.e., activations) of mPfc activity during a subsequent slow-wave sleep epoch (*match* epoch). It was found that (re)activations occurred preferentially during sharp wave/ripple complexes in post experience episodes, but not during previous sleep phases [35]. In another recent study, Benchenane et al. [36] reported that Pfc ensemble activations occur preferentially during periods of high theta coherence between the hippocampus and Pfc in a Y-maze task, which tended to occur during the decision point. These remarkable findings demonstrate that the use of PCA to estimate ensemble activity is a powerful tool to study network functioning. However, as illustrated in the present report, the framework applied in previous studies [32], [33], [35], [36] possibly merges the activity of multiple cell assemblies into a single activity pattern. In this sense, the extension of the method now introduced allows for the isolation of the activity patterns of distinct groups of neurons. We believe that sorting out the individual activity of different assemblies will provide important insights in future studies.

While the studies mentioned above have focused on a template matching approach, the results shown in Figure 8 were obtained by first identifying all cell assemblies present in the network and subsequently assessing their activity time course in the same time period used to identify them; notice therefore that the method can be employed in different ways. One should however be cautious to avoid potential spurious results derived from circular analysis [54] when using the template-match approach. For example, it will likely happen that assembly activity during the template epoch (in which the assemblies are defined) is higher than that of any other epoch not used for computing the activity projectors; therefore, we believe one should not make quantitative inferences about assembly activity during the template epoch compared to other epochs.

It is important to emphasize that the PCA-based method is not sensitive to sequences of neuronal activity, such as *synfire chains* [27], [55], [56]. As pointed in Peyrache et al. [34], the statistical difficulties accompanying methods that look for firing sequences are overwhelming when one needs to analyze larger networks [47], [49]. In fact, a common strategy to bypass combinatorial explosion (the number of possible temporal patterns is larger than the number of samples) is to detect ensemble activity disregarding the precise identity of the cell assemblies [57], [58], [59]. It is also important to note that only a tiny fraction of the neurons in the brain is observable, and therefore synfire chains are likely the effect of underlying sequences of cell assemblies, also known as Hebb's phase sequences [10]. The assessment of assembly sequences can be potentially achieved by the use of our method in combination with methods for detecting sequential activations [26], [50], [53].

It is also important to consider that the bin size used for the analyses can be critical for the interpretation of the results. As recently noted [12], bin sizes up to 30 ms are potentially well suited to analyze assembly activations. For instance, the typical membrane integration time in the waking cerebral cortex is estimated to vary between 10 and 30 ms [60], [61]. Moreover, previous work has shown that neuronal members of a putative cell assembly tend to synchronize transiently in time windows of approximately 25 ms [14], [62]. Interestingly, the time window for spike timing dependent plasticity is also consistent with this time-scale [63], [64], [65]. Finally, this time-scale corresponds to the period of gamma oscillations, which are believed to play a key role in binding representations coded by transiently active cell assemblies [66].

The novel framework described here allows the study of cell assemblies with shared neurons. The importance of this achievement is related to how information is processed and stored in the brain. Some authors suggest that each neuron would only fire to a specific concept or stimulus (grandmother cells) [67]; therefore, cell assemblies encoding different “things” would not be expected to share neurons. However, a mounting body of work shows that neurons can be very selective (*sparse* coding), but are not grandmother cells [68], [69]. The apparent grandmother cells in the human medial temporal lobe [70] may actually respond to between 50 and 150 distinct concepts [71]. Neurons participating in the representation of multiple concepts imply that the processing of information is distributed and occurs through a *multiplexed* code, in which concepts are represented by the activity of partially-overlapping groups of neurons, as postulated by Hebb.

Despite the worldwide acceptance of the cell assembly theory, there is still a paucity of evidence corroborating (or disproving) it. Hebb's hypotheses not only deal with the formation of assemblies and phase sequences, but also constitute a complete theory describing how learning, fear, hunger, and other complex behaviors emerge from the brain [1]. Most of the difficulty in testing the theory resides in the fact that only a tiny fraction of neurons in the brain can be simultaneously recorded at any given time. However, techniques for massive neuronal recordings are being developed at accelerating rates [22], and while we still lack proper tools for analyzing large quantities of neurons [57], [72], much progress is being made to circumvent this limitation. We hope the work presented here constitutes a useful step in this direction.

## Methods

Simulations and data analyses were programmed in MATLAB (The Mathworks, Inc); MATLAB codes for the computation of cell assemblies and their dynamics can be obtained from the authors upon request.

### Analytical formula of the Marčenko-Pastur distribution

The spectrum of eigenvalues of an autocorrelation matrix computed from a random matrix ***M*** of 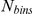 columns and 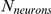 rows follow the Marčenko-Pastur distribution, which in the limit of 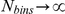 and 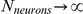, with 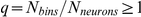 constant, is given by
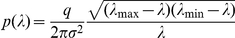
where 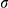 is the standard deviation of the elements of ***M*** (in our case, we have 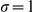 since we apply the z-score normalization to the binned spike activity); 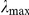 and 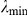 are the upper and lower limits of the Marčenko-Pastur distribution, and they are given by:



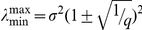
Notice that 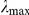 and 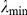 converge to 1 when 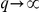 and in this limit the theoretical distribution becomes a Dirac delta function at 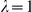. Therefore, the predicted eigenvalues distribution for independent neuronal activity has lower variance when a greater number of time bins are analyzed for a given number of neurons (compare Figure 3A and 3C).

We note that even though the analytical formula for the Marčenko-Pastur distribution was derived in the limit case of large 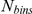 and 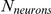, this theoretical distribution also approximates the actual distribution in cases of finite matrices, as shown in Plerou et al. [73] and in the present work. Nevertheless, one can also make use of the bias correction for finite size matrices suggested in [74]. The upper theoretical limit then becomes 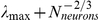. We found however that this correction did not influence the results shown in the present work.

### Outer product and the definition of the activity projector operators

The outer product of two vectors **u** and **v** of length *N* is defined as
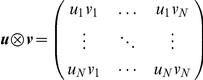
The outer product is used to construct the projectors of ensemble activity, as explained in the following. Let ***C*** be the autocorrelation matrix of a z-scored binned network activity ***Z*** of dimension 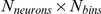, and let ***p_i_*** (*i = 1*,*2*,*…*, 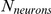) denote the principal components of ***C***. The projector ***P_i_*** associated with ***p_i_*** is given by
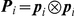
If 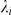 is the eigenvalue associated with the principal component ***p_i_***, ***C*** can be decomposed as
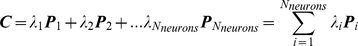
Assuming that each principal component ***p_i_*** represents an ensemble co-activation pattern, the equation above shows that ***C*** can be represented by a linear combination of the pattern representations encoded in the matrices ***P*** *_i_*.

### Assembly activity time-course

Peyrache et al. [34] has recently proposed the use of the principal components associated with significant eigenvalues for assessing ensemble activity with a single-bin resolution. The idea is to calculate the instantaneous similarity of the binned spike activity and the ensemble activity pattern as a function of time.

Let ***P*** be outer product of a significant principal component with itself and ***Z(b)*** be the *b*-*th* column of the z-scored binned spike activity (in other words, the number of spikes of all neurons in the *b*-*th* bin). The measure of instantaneous similarity of ***P*** and ***Z*** as a function of time is given by
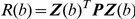
This equation can be rewritten as
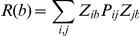
where *Z_ib_* is the normalized firing rate of neuron *i* in bin *b*, and *P_ij_* is the entry in the *i-th* row and *j-th* column of ***P***. Note that when *i* = *j*, the corresponding term of the summation only takes into account the activity of a single neuron *i*. Since our goal is to measure ensemble activity more than single neuron activations, this term can be set to zero and the equation reduces to
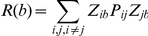
which is the equation for computing the time course of ensemble activity used in Peyrache et al. [34].

As we show in the present work (Figure 2 and S3), projectors computed as above are not appropriate to track the activity time course of individual cell assemblies if there are overlapping neurons among assemblies. To overcome this problem, we propose constructing the projectors using the optimal assembly vectors in Assembly space (Figures 6 and 7). This is achieved as follows: The Assembly space is defined as the metric subspace spanned by the principal components ***p_i_*** associated with eigenvalues *λ_i_* that are significantly above chance. Let ***a*** *_k_* (*k* = *1*,*…*, 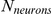) denote the neuron vectors in the Assembly space; each ***a*** *_k_* is given by (see Figure 6E):
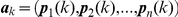
where *n* is the number of significant eigenvalues. As we show in the present work, the number of eigenvalues outside the theoretical distribution gives the total number of neurons participating in cell assemblies. Supposing there are 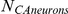 assembly neurons, they correspond to the 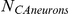 vectors with largest norm (vector length) in Assembly space (Figures 6E and 7E). Then, the projections of each neuron vector in the Assembly space onto the direction of the other vectors are computed and used to build the Interaction Matrix (Figures 6F and 7F). That is, given two neuron vectors ***a*** *_i_* and ***a*** *_j_*, the corresponding (*i*,*j*) entry of the Interaction Matrix is given by (***a*** *_i_*•***a*** *_j_*)/(***a*** *_j_*•***a*** *_j_*). From the Interaction Matrix, it is possible to determine which neurons compose each assembly by means of a clustering algorithm (see next section). The estimated optimal assembly vector 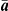 is then defined for an assembly A as the mean over ***a*** *_i_*'s for all neurons *i* exclusive to A, normalized to have unitary norm:
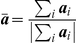
Next, 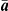 is expressed as a linear combination of the significant principal components:
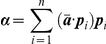
A projector 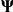 is then calculated as the outer product of 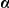 with itself (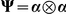). Finally, we use 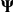 to compute the activity time course of assembly A as follows:
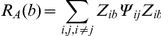



### Binary Interaction Matrix and clustering algorithm

The algorithm identifies the neurons pertaining to each cell assembly based on the analysis of the Interaction Matrix. The entries of the Interaction Matrix are a measure of correlation between two neuron vectors in Assembly space (taking into account only cell assembly neurons). As we have shown in Figures 6 and 7, neurons that pertain to different assemblies are orthogonal to each other, while high collinearity levels indicate that neurons are correlated in the Assembly space. Therefore, it is expected that the distribution of Interaction Matrix entries is bimodal, having sets of low and high values (see Figure S1). We then apply a uni-dimensional version of the K-means clustering algorithm [75] in order to find a threshold that best separate these groups. We use this threshold to create a binary Interaction Matrix; that is, we transform all matrix values in 0's (values below a threshold) and 1's (values above the threshold). This binary matrix is the input to the clustering algorithm which is then able to sort apart the neurons of different assemblies. In Figure S1 we provide an overview of the thresholding procedure and in Figure S2 we describe the clustering algorithm.

### Electrophysiological recordings

Male Long-Evans rats were chronically implanted with tungsten microelectrode arrays aimed at the hippocampus, primary visual cortex and primary somatosensory cortex. Data recorded from these animals were described in a previous study [44], in which a detailed description of surgery, data collection, behavior and histology can be found.

## Supporting Information

Figure S1 Interaction Matrix thresholding. (**A**) Ai: Interaction Matrix of the example shown in Figure 7. Aii: Histogram of the entries of the Interaction Matrix shown in Ai. Dashed red line indicates the threshold found by a K-means algorithm. The threshold is the mean between the borders of the clusters. Aiii: Binary Interaction Matrix. Values lower and higher than the threshold are set to 0 and 1, respectively. This matrix is later used as input to the clustering algorithm described in Figure S2. (**B**) Same as (**A**) but for the real data shown in Figure 8B. Note that the threshold found separates the bimodal distribution.
(TIF)

Figure S2 Description of the assembly clustering algorithm. (**A,B**) Flux diagram representing the three main steps of the algorithm (**A**) and an example using simulated data of nine neurons (**B**). The algorithm receives as input a Binary Interaction Matrix (BIM; depicted in **B** top panel), which is obtained by thresholding the Interaction Matrix (see Figure S1), and provides as output the assembly label(s) for each neuron (**B** bottom panel). Step 1 involves re-organizing the BIM according to the number of interactions in each row and also removing repeated rows; we denote the resulting matrix as the OBIM (**B** second panel from top). Notice in **B** that row #8 does not appear in the OBIM since it was equal to row #5. In Step 2 assembly labels are created and assigned to the neurons. This is achieved based on sequentially examining each row of OBIM and identifying for each neuron (row) all other neurons that interact with it; a common assembly label is ascribed to all interacting neurons. New assembly labels are created whenever the neuron (row) being processed has not been previously assigned to any of the existing assembly labels. This step generates the Assembly Label Matrix (ALM), which entry (*i*,*j*) informs the assemblies shared by neurons #*i* and #*j*. Notice that neuron #8 automatically appears in ALM under this procedure (**B** third panel from top). Finally, in Step 3 the assembly labels in the diagonal of ALM are extracted; they indicate the assemblies in which each neuron participates.
(TIF)

Figure S3 Estimation of time course of cell assembly activity based on individual PCs for the examples shown in Figures 6 (**A**) and 7 (**B**). The estimation of assembly activity based on assembly vectors is also reproduced from Figures 6 and 7 for comparison.
(TIF)

## References

[1] Hebb DO (1949). The Organization of Behavior.

[2] Wang Q, Chen G, Perc M (2011). Synchronous bursts on scale-free neuronal networks with attractive and repulsive coupling.. PLoS One.

[3] Wang Q, Perc M, Duan Z, Chen G (2009). Synchronization transitions on scale-free neuronal networks due to finite information transmission delays.. Phys Rev E Stat Nonlin Soft Matter Phys.

[4] Pikovsky A, Rosenblum M, Kurths J (2001). Synchronization, A Universal Concept in Nonlinear Sciences Cambridge University Press..

[5] Singer W (1993). Synchronization of cortical activity and its putative role in information processing and learning.. Annu Rev Physiol.

[6] Freiwald WA, Kreiter AK, Singer W (2001). Synchronization and assembly formation in the visual cortex.. Prog Brain Res.

[7] Volman V, Perc M (2010). Fast random rewiring and strong connectivity impair subthreshold signal detection in excitable networks.. New J Phys.

[8] Marr D (1971). Simple memory: a theory for archicortex.. Philos Trans R Soc Lond B Biol Sci.

[9] Wallenstein GV, Eichenbaum H, Hasselmo ME (1998). The hippocampus as an associator of discontiguous events.. Trends Neurosci.

[10] Harris KD (2005). Neural signatures of cell assembly organization.. Nature Reviews Neuroscience.

[11] Pastalkova E, Itskov V, Amarasingham A, Buzsaki G (2008). Internally generated cell assembly sequences in the rat hippocampus.. Science.

[12] Buzsaki G (2010). Neural syntax: cell assemblies, synapsembles, and readers.. Neuron.

[13] Wilson MA, McNaughton BL (1993). Dynamics of the hippocampal ensemble code for space.. Science.

[14] Harris KD, Csicsvari J, Hirase H, Dragoi G, Buzsaki G (2003). Organization of cell assemblies in the hippocampus.. Nature.

[15] Carmena JM, Lebedev MA, Crist RE, O'Doherty JE, Santucci DM (2003). Learning to control a brain-machine interface for reaching and grasping by primates.. Plos Biology.

[16] Hung CP, Kreiman G, Poggio T, DiCarlo JJ (2005). Fast readout of object identity from macaque inferior temporal cortex.. Science.

[17] Nicolelis MA, Ghazanfar AA, Stambaugh CR, Oliveira LM, Laubach M (1998). Simultaneous encoding of tactile information by three primate cortical areas.. Nat Neurosci.

[18] Wessberg J, Stambaugh CR, Kralik JD, Beck PD, Laubach M (2000). Real-time prediction of hand trajectory by ensembles of cortical neurons in primates.. Nature.

[19] Nicolelis MA, Fanselow EE, Ghazanfar AA (1997). Hebb's dream: the resurgence of cell assemblies.. Neuron.

[20] Nicolelis MAL, De Schutter E (1999). Methods for recording and analyzing neuronal ensemble activity.. Journal of Neuroscience Methods.

[21] Buzsaki G (2004). Large-scale recording of neuronal ensembles.. Nature Neuroscience.

[22] Stevenson IH, Kording KP (2011). How advances in neural recording affect data analysis.. Nat Neurosci.

[23] Lee AK, Wilson MA (2002). Memory of sequential experience in the hippocampus during slow wave sleep.. Neuron.

[24] Ribeiro S, Gervasoni D, Soares ES, Zhou Y, Lin SC (2004). Long-lasting novelty-induced neuronal reverberation during slow-wave sleep in multiple forebrain areas.. Plos Biology.

[25] Louie K, Wilson MA (2001). Temporally structured replay of awake hippocampal ensemble activity during rapid eye movement sleep.. Neuron.

[26] Abeles M, Gat I (2001). Detecting precise firing sequences in experimental data.. Journal of Neuroscience Methods.

[27] Ikegaya Y, Aaron G, Cossart R, Aronov D, Lampl I (2004). Synfire chains and cortical songs: Temporal modules of cortical activity.. Science.

[28] Maldonado P, Babul C, Singer W, Rodriguez E, Berger D (2008). Synchronization of neuronal responses in primary visual cortex of monkeys viewing natural images.. J Neurophysiol.

[29] Grun S, Diesmann M, Aertsen A (2002). Unitary events in multiple single-neuron spiking activity: 1. Detection and significance.. Neural Computation.

[30] Pipa G, Wheeler DW, Singer W, Nikolic D (2008). NeuroXidence: reliable and efficient analysis of an excess or deficiency of joint-spike events.. Journal of Computational Neuroscience.

[31] Chapin JK, Nicolelis MA (1999). Principal component analysis of neuronal ensemble activity reveals multidimensional somatosensory representations.. J Neurosci Methods.

[32] Nicolelis MAL, Baccala LA, Lin RCS, Chapin JK (1995). Sensorimotor encoding by synchronous neural ensemble activity at multiple levels of the somatosensory system.. Science.

[33] Humphries MD (2011). Spike-train communities: finding groups of similar spike trains.. J Neurosci.

[34] Peyrache A, Benchenane K, Khamassi M, Wiener SI, Battaglia FP (2010). Principal component analysis of ensemble recordings reveals cell assemblies at high temporal resolution.. Journal of Computational Neuroscience.

[35] Peyrache A, Khamassi M, Benchenane K, Wiener SI, Battaglia FP (2009). Replay of rule-learning related neural patterns in the prefrontal cortex during sleep.. Nature Neuroscience.

[36] Benchenane K, Peyrache A, Khamassi M, Tierney PL, Gioanni Y (2010). Coherent Theta Oscillations and Reorganization of Spike Timing in the Hippocampal-Prefrontal Network upon Learning.. Neuron.

[37] Theiler J, Eubank S, Longtin A, Galdrikian B, Doyne Farmer J (1992). Testing for nonlinearity in time series: the method of surrogate data.. Physica D: Nonlinear Phenomena.

[38] Schreiber T, Schmitz A (1996). Improved Surrogate Data for Nonlinearity Tests.. Physical Review Letters.

[39] Perc M, Green AK, Dixon CJ, Marhl M (2008). Establishing the stochastic nature of intracellular calcium oscillations from experimental data.. Biophys Chem.

[40] Pradhan N, Sadasivan PK (1996). Relevance of surrogate-data testing in electroencephalogram analysis.. Phys Rev E Stat Phys Plasmas Fluids Relat Interdiscip Topics.

[41] Khoa TQD, Thang HM, Nakagawa M (2008). Testing for Nonlinearity in Functional Near-Infrared Spectroscopy of Brain Activities by Surrogate Data Methods.. The Journal of Physiological Sciences.

[42] Schreiber T (1999). Interdisciplinary application of nonlinear time series methods.. Physics Reports.

[43] Marčenko VA, Pastur LA (1967). Distribution of eigenvalues for some sets of random matrices.. Mathematics of the USSR-Sbornik.

[44] Ribeiro S, Shi X, Engelhard M, Zhou Y, Zhang H (2007). Novel experience induces persistent sleep-dependent plasticity in the cortex but not in the hippocampus.. Front Neurosci.

[45] Schneidman E, Berry MJ, Segev R, Bialek W (2006). Weak pairwise correlations imply strongly correlated network states in a neural population.. Nature.

[46] Berger D, Warren D, Normann R, Arieli A, Grun S (2007). Spatially organized spike correlation in cat visual cortex.. Neurocomputing.

[47] Mokeichev A, Okun M, Barak O, Katz Y, Ben-Shahar O (2007). Stochastic emergence of repeating cortical motifs in spontaneous membrane potential fluctuations in vivo.. Neuron.

[48] Berger D, Borgelt C, Louis S, Morrison A, Grun S (2010). Efficient identification of assembly neurons within massively parallel spike trains.. Comput Intell Neurosci.

[49] Grun S (2009). Data-Driven Significance Estimation for Precise Spike Correlation.. Journal of Neurophysiology.

[50] Tetko IV, Villa AEP (2001). A pattern grouping algorithm for analysis of spatiotemporal patterns in neuronal spike trains. 1. Detection of repeated patterns.. Journal of Neuroscience Methods.

[51] Abeles M, Gerstein GL (1988). Detecting spatiotemporal firiging patterns amog simultaneously recorded single neurons.. Journal of Neurophysiology.

[52] Wilson MA, McNaughton BL (1994). Reactivation of hippocampal ensemble memories during sleep.. Science.

[53] Shmiel T, Drori R, Shmiel O, Ben-Shaul Y, Nadasdy Z (2006). Temporally precise cortical firing patterns are associated with distinct action segments.. Journal of Neurophysiology.

[54] Kriegeskorte N, Simmons WK, Bellgowan PSF, Baker CI (2009). Circular analysis in systems neuroscience: the dangers of double dipping.. Nature Neuroscience.

[55] Prut Y, Vaadia E, Bergman H, Haalman I, Slovin H (1998). Spatiotemporal structure of cortical activity: Properties and behavioral relevance.. Journal of Neurophysiology.

[56] Abeles M (1991). Corticonics: Neural Circuits of the Cerebral Cortex: Cambridge University Press, New-York..

[57] Louis S, Borgelt C, Grun S (2010). Complexity distribution as a measure for assembly size and temporal precision.. Neural Networks.

[58] Staude B, Rotter S, Grun S (2010). CuBIC: cumulant based inference of higher-order correlations in massively parallel spike trains.. Journal of Computational Neuroscience.

[59] Staude B, Grun S, Rotter S (2010). Higher-order correlations in non-stationary parallel spike trains: statistical modeling and inference.. Frontiers in Computational Neuroscience.

[60] Koch C, Rapp M, Segev I (1996). A brief history of time (constants).. Cerebral Cortex.

[61] Leger JF, Stern EA, Aertsen A, Heck D (2005). Synaptic integration in rat frontal cortex shaped by network activity.. Journal of Neurophysiology.

[62] Kelemen E, Fenton AA (2010). Dynamic Grouping of Hippocampal Neural Activity During Cognitive Control of Two Spatial Frames.. Plos Biology.

[63] Bi GQ, Poo MM (1998). Synaptic modifications in cultured hippocampal neurons: Dependence on spike timing, synaptic strength, and postsynaptic cell type.. Journal of Neuroscience.

[64] Markram H, Lubke J, Frotscher M, Sakmann B (1997). Regulation of synaptic efficacy by coincidence of postsynaptic APs and EPSPs.. Science.

[65] Levy WB, Steward O (1983). Temporal contiguity requirements for long-term associative potentiation depression in the hippocampus.. Neuroscience.

[66] Fries P, Nikolic D, Singer W (2007). The gamma cycle.. Trends Neurosci.

[67] Bowers JS (2009). On the biological plausibility of grandmother cells: implications for neural network theories in psychology and neuroscience.. Psychol Rev.

[68] Quiroga RQ, Kreiman G, Koch C, Fried I (2008). Sparse but not ‘Grandmother-cell’ coding in the medial temporal lobe.. Trends in Cognitive Sciences.

[69] Gelbard-Sagiv H, Mukamel R, Harel M, Malach R, Fried I (2008). Internally generated reactivation of single neurons in human hippocampus during free recall.. Science.

[70] Quiroga RQ, Reddy L, Kreiman G, Koch C, Fried I (2005). Invariant visual representation by single neurons in the human brain.. Nature.

[71] Waydo S, Kraskov A, Quiroga RQ, Fried I, Koch C (2006). Sparse representation in the human medial temporal lobe.. Journal of Neuroscience.

[72] Brown EN, Kass RE, Mitra PP (2004). Multiple neural spike train data analysis: state-of-the-art and future challenges.. Nature Neuroscience.

[73] Plerou V, Gopikrishnan P, Rosenow B, Amaral LAN, Guhr T (2002). Random matrix approach to cross correlations in financial data.. Physical Review E.

[74] Tracy CA, Widom H (1994). Level-spacing distributions and the airy kernel.. Communications in Mathematical Physics.

[75] Lloyd SP (1982). Least-squares quantizatoin in PCM.. Ieee Transactions on Information Theory.

