# Supplementary figures and images for: Neuronal Assembly Detection and Cell Membership Specification by Principal Component Analysis
Source: PLoS One. 2011 Jun 15;6(6):e20996. doi: 10.1371/journal.pone.0020996 (PMC3115970; doi:10.1371/journal.pone.0020996)

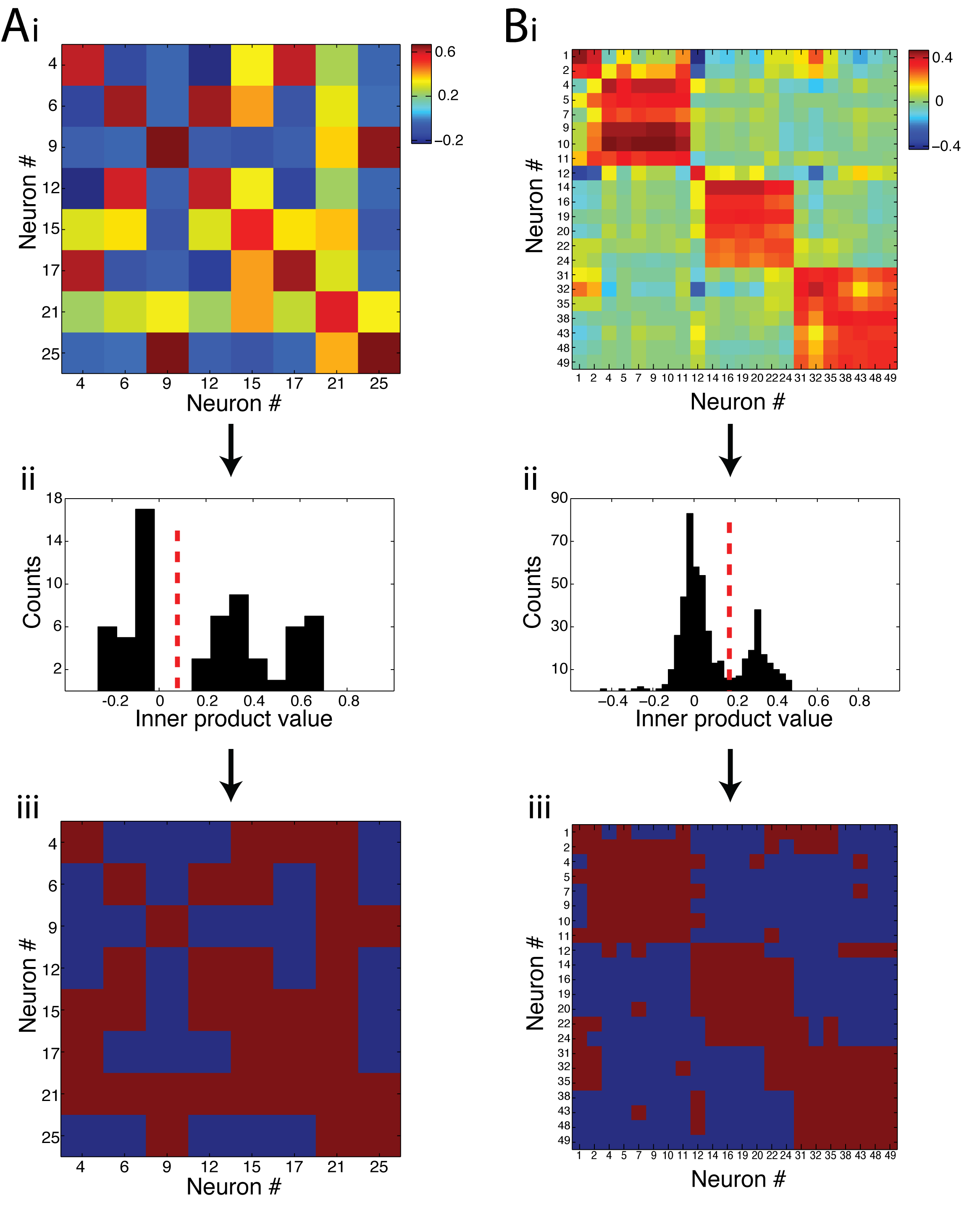

Supplement: Figure S1 — Interaction Matrix thresholding. (A) Ai: Interaction Matrix of the example shown in Figure 7. Aii: Histogram of the entries of the Interaction Matrix shown in Ai. Dashed red line indicates the threshold found by a K-means algorithm. The threshold is the mean between the borders of the clusters. Aiii: Binary Interaction Matrix. Values lower and higher than the threshold are set to 0 and 1, respectively. This matrix is later used as input to the clustering algorithm described in Figure S2. (B) Same as (A) but for the real data shown in Figure 8B. Note that the threshold found separates the bimodal distribution. (TIF) [file pone.0020996.s001.tif]

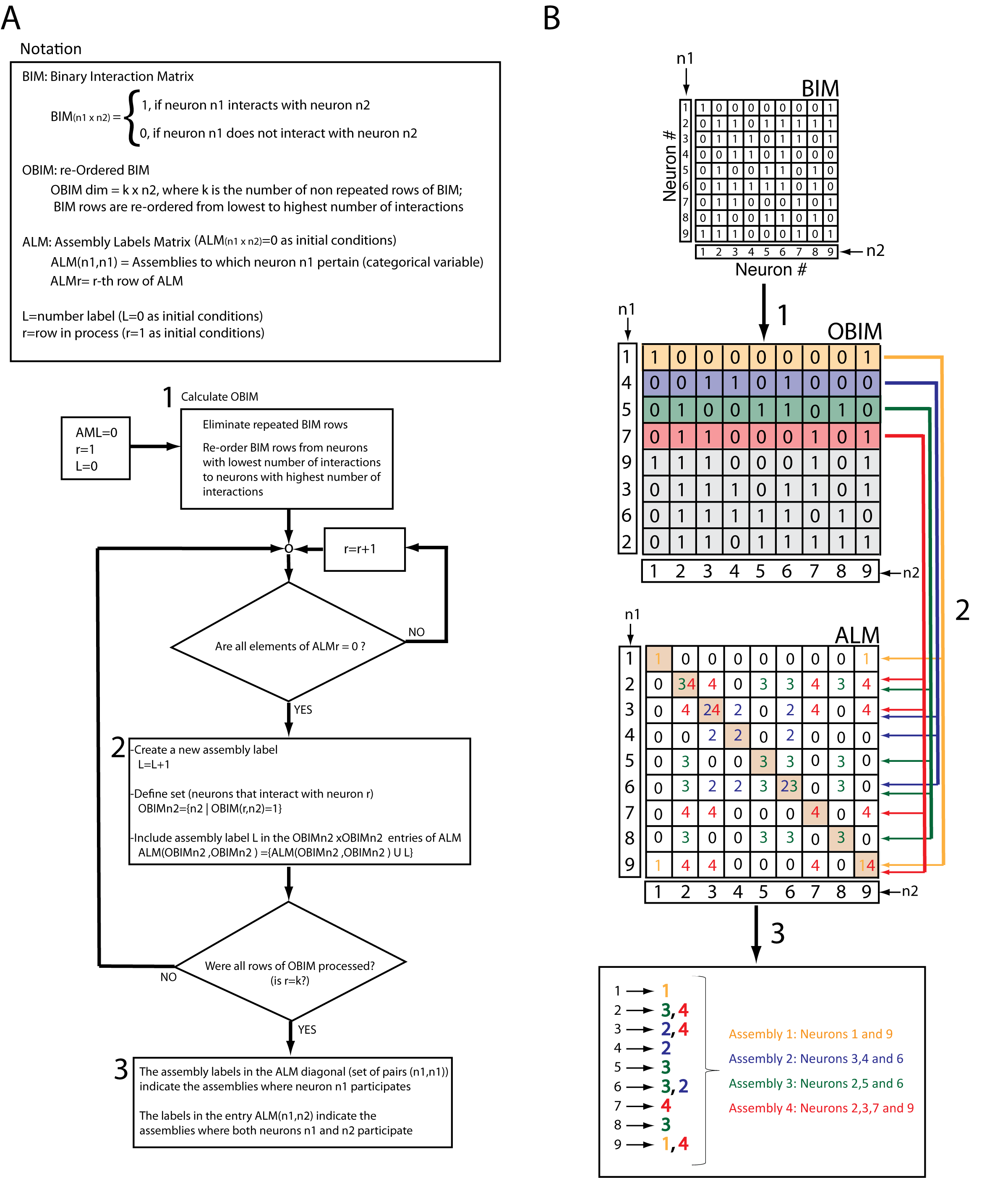

Supplement: Figure S2 — Description of the assembly clustering algorithm. (A,B) Flux diagram representing the three main steps of the algorithm (A) and an example using simulated data of nine neurons (B). The algorithm receives as input a Binary Interaction Matrix (BIM; depicted in B top panel), which is obtained by thresholding the Interaction Matrix (see Figure S1), and provides as output the assembly label(s) for each neuron (B bottom panel). Step 1 involves re-organizing the BIM according to the number of interactions in each row and also removing repeated rows; we denote the resulting matrix as the OBIM (B second panel from top). Notice in B that row #8 does not appear in the OBIM since it was equal to row #5. In Step 2 assembly labels are created and assigned to the neurons. This is achieved based on sequentially examining each row of OBIM and identifying for each neuron (row) all other neurons that interact with it; a common assembly label is ascribed to all interacting neurons. New assembly labels are created whenever the neuron (row) being processed has not been previously assigned to any of the existing assembly labels. This step generates the Assembly Label Matrix (ALM), which entry (i,j) informs the assemblies shared by neurons #i and #j. Notice that neuron #8 automatically appears in ALM under this procedure (B third panel from top). Finally, in Step 3 the assembly labels in the diagonal of ALM are extracted; they indicate the assemblies in which each neuron participates. (TIF) [file pone.0020996.s002.tif]

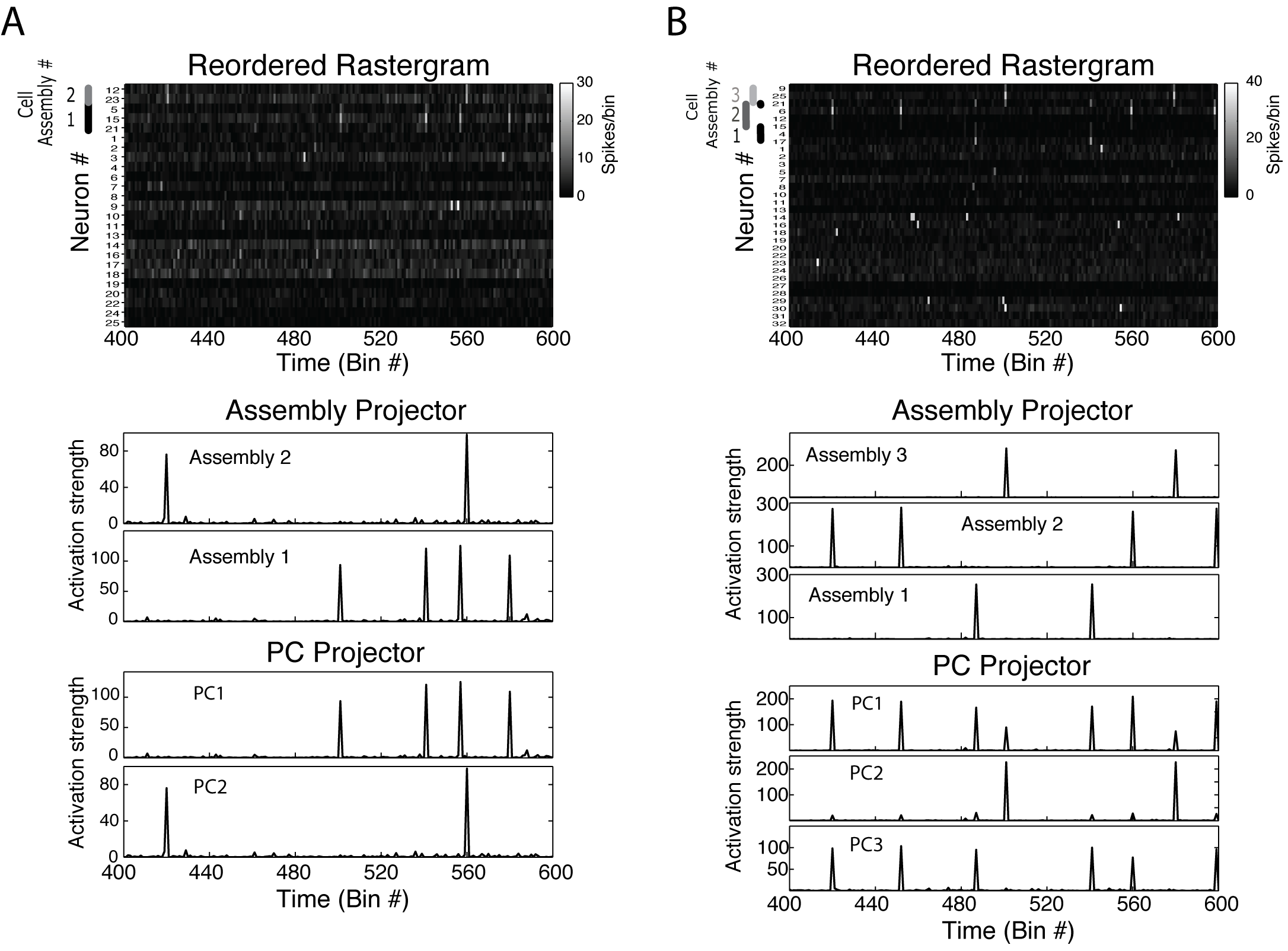

Supplement: Figure S3 — Estimation of time course of cell assembly activity based on individual PCs for the examples shown in Figures 6 (A) and 7 (B). The estimation of assembly activity based on assembly vectors is also reproduced from Figures 6 and 7 for comparison. (TIF) [file pone.0020996.s003.tif]
